# Metabolome analysis of key genes for synthesis and accumulation of triterpenoids in *Wolfiporia cocos*

**DOI:** 10.1038/s41598-022-05610-3

**Published:** 2022-01-28

**Authors:** GuiPing Zeng, Zhong Li, Zhi Zhao

**Affiliations:** 1 Guizhou University, Guiyang, 550025 China; 2 Guizhou Key Laboratory of Propagation and Cultivation On Medicinal Plants, Guizhou University, Guiyang, 550025 China

**Keywords:** Biochemistry, Microbiology, Physiology

## Abstract

Triterpenoid, the active ingredient in the dried sclerotia of *Wolfiporia cocos*, has a variety of pharmacological effects. The focus of this research was the cell engineered bacteria modified for triterpenoid biosynthesis, and we aimed to identify the key genes involved in triterpenoid biosynthesis and their roles. Two monospora strains, H and L, were selected from the sexually propagated progeny of *W. coco* strain 5.78, and their mycelia were cultured for 17, 34, and 51 days. Metabolite analysis showed that there were significantly more down-regulated metabolites of the two strains at three different culture periods than up-regulated metabolites. KEGG indicated that the differential metabolites were mainly concentrated in sterol biosynthesis and ABC transport. STEM analysis suggested that polysaccharide synthesis and accumulation might be greater in the L strain than the H strain. The correlation analysis of DEGs and differential metabolites between the two strain groups showed that *erg11* and *FDPS*, which were closely positively correlated with differential metabolites associated with triterpenoids, were highly expressed in the L strain. This result suggested that the high expression of some genes in the L strain might shunt precursor substances of triterpenoids, which was the possible reason for the decrease in the synthesis and accumulation of triterpenoids.

## Introduction

A traditional Chinese medicine, the dried sclerotia of *Wolfiporia cocos* (Schwein.) Ryvarden and Gilb., has a history dating back more than 2000 years. The main active components of *W. cocos* are polysaccharides and triterpenoids^1^, of which polysaccharides (β-pachyman) and triterpenoids (Lanostane type and 3, 4-cyclolanolin types) are the main chemical components^2^. In the past two decades, researchers in Japan and China have isolated almost all triterpenoids from *W. cocos*, which are derivatives of lanosterol or secondary lanosterol^1^. Triterpene saponins are secondary metabolites of plants and participate in the regulation of plant communication, defense, and sensory functions^3^. Modern pharmacology studies have shown that triterpenoids in *W. cocos* have antitumor^4,5^, enhanced immunity^6,7^, reduced blood sugar^8,9^, antioxidant^10^, anti-inflammatory^11,12^, kidney disease treatment^13,14^, diuretic^15,16^, hepatoprotective^17,18^, antiemetic^19^, anticonvulsant^20^, and whitening^21^ effects, among others. Therefore, triterpenoids in *W. cocos* have an extensive and important role in future medical applications.

*W. cocos* is an aerobic saprophytic fungus which is parasitic in the roots of pine trees such as *Pinus densiflora* and *Pinus massoniana Lamb.*. The demand for large scale development of *W. cocos* conflicts with the protection of pine resources. The wood resource problem has become the main bottleneck in the sustainable development of *W. cocos*. Industrial production of triterpenoids by fermenting hyphae has practical significance in terms of speed, efficiency, and environmental protection. Among the factors affecting fermentation efficiency, the acquisition of superior strains and the expression of key genes of triterpenoid biosynthesis and their regulatory mechanism are problems that need to be solved urgently.

Following genomics, transcriptome and proteomics, metabonomics is a newly developed discipline. It takes metabolite molecules in biological systems as the object, and uses modern instrumental analysis methods with high throughput, sensitivity and resolution, combined with pattern recognition and other stoichiometric methods, to analyze the change in metabolites over time after stimulation or disturbance of the biological system. Since the qualitative and quantitative conditions of all metabolites in an organism are examined, the metabolome can comprehensively reflect the metabolic conditions of the biological system under study.

Our research team obtained genes related to the high accumulation of triterpenoids through transcriptome analysis, and in this study we aim to identify the key genes related to the synthesis and accumulation of triterpenoids through metabonomics analysis combined with transcriptome analysis. The results will provide a theoretical basis for regulating the production of triterpenoids by fermentation.

## Materials and methods

### Biomaterials and culture methods

Both the high-yielding DZAC-Wp-H-29 (H) and low-yielding DZAC-Wp-L-123 (L) triterpenoid strains were derived from the sexually reproduced progeny strain 5.78 of *W. cocos* (purchased from the Institute of Microbiology, Chinese Academy of Sciences, Beijing, China, and stored in a refrigerator at − 80 °C at the Institute of Fungal Resources, Guizhou University)^22^. Experimental research and field studies on plants were conducted in compliance with relevant institutional, national, and international guidelines and legislation in the methods section. For the *W. cocos* potato dextrose agar (PDA) medium (no. 17 medium, Institute of Microbiology, Chinese Academy of Sciences), potatoes were washed, peeled, and cut into pieces, and 200 g of potatoes were put into 1000 mL of water, boiled for 30 min, and filtered by gauze. The filtrate was mixed with 1000 mL distilled water with 20 g glucose, 1 g KH_2_PO_4_, 0.5 g MgSO_4_•7H_2_O, 10 mg VB_1_, and 18 g agar at natural pH. Mycelia were cultured for 17, 34, and 51 d at 25 °C in the dark, with 6 replicates that were quickly frozen in liquid nitrogen, then stored in a refrigerator at − 80 °C^22^.

### Extraction and detection of metabolites

1. Standard products and reagents

1. Methanol (Lot #1230, LCMS grade, CAS: 67–56-1) was purchased from CNW Technologies (ANPEL Laboratory Technologies (Shanghai) Inc.);
2. Acetonitrile (Lot #1646, LCMS grade, CAS: 75–05-8) was purchased from CNW Technologies (ANPEL Laboratory Technologies (Shanghai) Inc.);
3. Water was provided by a pure water meter;
4. Ammonium acetate (LOT #1350K100, LCMS, CAS: 631–61-8) was purchased from CNW Technologies (ANPEL Laboratory Technologies (Shanghai) Inc.);
5. Ammonium hydroxide (LCMS级, CAS: 1336–21-6)was purchased from CNW Technologies (ANPEL Laboratory Technologies (Shanghai) Inc.);
6. Internal standard: L-2-chloro-L-phenylalanine (CAS: 103616–89-3, purity ≥ 98%) was purchased from Shanghai Hengbai Biotechnology Co., Ltd., China.

2. Instrument platform.

1. Ultra-high performance liquid phase: Agilent 1290 UHPLC, Agilent;
2. High Resolution Mass Spectrometry: Q ExActive Orbitrap (Thermo Fisher Scientific, USA);
3. Chromatographic column: ACQUITY UPLC HSS T3 1.7 μm 2.1 * 100 mm, Waters;
4. Grinding instrument: JXFSTPRP-24, Shanghai Jingxin Technology Co., Ltd;
5. Centrifuge: Thermo Scientific Heraeus Fresco17 centrifuge;
6. Vortex instrument: Vortex 5, Krymbel Instrument Manufacturing Co., Ltd;
7. Balance: BSA124S-CW, Sartorius Scientific Instruments (Beijing) Co., Ltd;
8. Pure water instrument: Mingche D24 UV, Merck Millipore, Germany;
9. Ultrasonic instrument: PS-60AL, Shenzhen Redbon Electronics Co., Ltd., China.

3. Extraction of metabolites.

Each 100 μg sample was homogenized and ground to a powder, and 300 μL methanol and 20 μL internal standard were added. After eddy mixing for 30 s, ultrasonic extraction was carried out in an ice water bath for 5 min, and the sample was then kept at –20 ℃ for 2 h, centrifuged at 13,000 rpm at 4 ℃ for 15 min, and 200 μL supernatant was put into a 2 mL injection flask. Liquid chromatograph-mass spectrometer (LC–MS) analysis was then performed.

Quality control (QC) samples were prepared by mixing the extract of the experimental samples in equal amounts to analyze the repeatability of the samples under the same treatment method. During instrumental analysis, one quality control sample was inserted in every 6–10 tests and analysis samples to monitor the repeatability of the analysis process.

4. Metabolite detection.

The instrument platform for LC–MS analysis consisted of the Agilent 1290 ultra-performance liquid chromatography in tandem with Thermo Fisher Scientific's Q ExActive Orbitrap High Resolution Mass Spectrometer. The chromatographic column was UPLC HSS T3 (1.7 μm 2.1 * 100 mm, Waters).

Mobile phase conditions (Table 1):

**Table 1:** Condition parameters of mobile phase (sample volume: 1 μL).

Time (min)	Row (μL/min)	A%	B%
0	500	99	1
1.0	500	99	1
8.0	500	1	99
10.0	500	1	99
10.1	500	99	1
12.0	500	99	1

1. Positive mode: mobile phase A: 0.1% formic acid aqueous solution;
2. Mobile phase B: acetonitrile;
3. Negative mode: mobile phase A: 5 mM ammonium acetate aqueous solution (adjust pH to 9.0 with ammonia water);
4. Mobile phase B: acetonitrile.

A Q ExActive Orbitrap high resolution mass spectrometer was used to collect the primary and secondary mass spectrum data.

The parameter conditions of the mass spectrum were as follows: The ESI ion source spray voltage was 3800 V in positive ion mode and –3100 V in negative ion mode; the capillary temperature was 320 ℃; the sheath gas flow rate was 45 Arb; the auxiliary gas flow rate was 15 Arb; the scanning range was 70–1000 m/z; level 1 resolution was set at 70,000 and the secondary resolution was 17,500; the intensity of step-by-step collision energy was 3; stepwise collision energy was 20, 40, and 60 eV; and the scanning rate was 7 Hz.

We selected the QC sample by the Fullms DDMS2 method, and set the following scan sections according to the following ranges:

1. 70–200,
2. 190–400,
3. 390–600,
4. 590–1000,

Each scan segment is collected once to obtain more secondary data for identification.

### Metabolome data analysis

1. Pre-processing of original data.

The original data after mass spectrometry analysis were first converted into MZML format by ProteoWizard software. Then, XCMS was used for retention time correction, peak recognition, peak extraction, peak integration, peak alignment, and normalization. Finally, a data matrix of the retention time, mass/charge ratio, and peak strength was obtained.

2. Metabolite identification and quantification.

The data were filtered using the filtering standard to retain the substance if the sample number of the substance was detected in all the samples at ≥ 50% (minifrac = 0.5). OSI-SMMS software (version No. : V1.0, Dalian Dashuo Information Technology Co., Ltd.) was used to characterize the metabolites according to the public database and the secondary database established by the software.

3. Multivariate statistical analysis.

The normalized data matrix was used for multivariate statistical analysis, including principal component analysis (PCA) , partial least square discriminant analysis (PLS-DA), and orthogonal partial least squares discriminant analysis (OPLS-DA). PLS-DA^23^ is a multivariate statistical analysis method with supervised pattern recognition. Before compression, multi-dimensional data are grouped according to the difference factors that need to be looked for (the Y value is set in advance for target classification and discrimination), so that the variables that are most relevant to the factors for grouping can be identified, and the influence of some other factors can be reduced. Similar to PCA analysis, PLS-DA maximizes the differentiation between groups and facilitates the search for differential metabolites. Variable importance in projection (VIP) of the model variables can be used to measure the impact and explanatory ability of different metabolite accumulation differences on the classification discrimination of each group. VIP ≥ 1 is the common screening criterion for different metabolites. OPLS-DA analysis was adopted in this study, which is an algorithm derived from PLS-DA. Compared with PLS-DA, OPLS-DA combines two methods of orthogonal signal correction (OSC) and PLS-DA, which can decompose the X matrix into two types of information: Related to Y and unrelated to Y. By removing the unrelated differences, relevant information is concentrated in the first prediction component.

A ranking test is a random ranking method used to evaluate the accuracy of OPLS models. It is used to avoid the classification of supervised learning methods that is not accidental. In this method, the X matrix is fixed, and the variables (e.g., 0 and 1) of the classification Y matrix defined previously are randomly arranged N times (generally 100–1000 times). After each arrangement and combination, a new (O) PLS model was constructed, and the R2 and Q2 values accumulated by the corresponding model were calculated. The criterion for evaluating model quality was as follows: The rightmost two points (x = 1.0) were R2 and Q2 of the original model, and all points on the left were R2' and Q2' of the model after Y replacement. If these R2' and Q2' are less than the original R2 and Q2, the model is considered meaningful.

4. Differential metabolite identification.

Multivariate statistical analysis of VIP values of OPLS-DA and univariate statistical analysis of T test (T-test) P values were used to identify metabolites with significant differences between groups^24^. Results were considered statistically significant with VIP ≥ 1 and P < 0.05.

5. KEGG enrichment analysis of metabolic pathway.

After the metabolites were found, the differential metabolites were compared to Kyoto Encyclopedia of Genes and Genomes (KEGG) database^25^ (http://www.genome.jp/kegg) for metabolite enrichment pathway analysis, and the hypergeometric test was applied to find out the significant enrichment pathway in the differential metabolites compared with the background of all metabolites. After the calculated P value is corrected by false discovery rate (FDR), the corrected *P* value ≤ 0.05 is taken as the threshold, and the pathway that satisfies this condition is defined as the pathway that is significantly enriched in the candidate metabolites.

6. STEM analysis of metabolites.

Short Time-series Expression Miner (STEM)^26^ software was used to load the files of the expression levels of all differential metabolites (the samples were sequenced according to biological logic). To examine the expression pattern of differential metabolites, the expression data from each sample (in the order of treatment) were normalized to 0, log_2_ (v1/v0), log_2_ (v2/v0), and then clustered by STEM. The parameters -pro 8 and ratio 1.0 (default settings for other parameters were used) were selected for trend analysis. The P value of each trend was calculated by hypothesis test, with statistical significance set at P ≤ 0.05. The trend block satisfying this condition was defined as the trend of significance.

7. Association analysis of differential metabolites and differential genes.

Hd17-VS-Ld17, Hd34-VS-Ld34, and Hd51-VS-Ld51 were selected between groups. Due to the different number of replicates among groups, the mean values of differential metabolites (6 replicates) and differentially expressed genes (3 replicates) were used to calculate the correlation coefficient, and the P value was calculated. The threshold value of r > 0.9 and P < 0.01 was used to screen the differential genes and metabolite pairs, and was plotted by Cytoscape.

### Statistical analysis

SPSS statistics software was used for significance test and other basic calculations. The principal component and correlation analyses were conducted with the R-language package (R 3.5.0 2018) (http://www.r-project.org/). Select the default parameters to run. Graph Pad Prism7.0 (https://www.graphpad.com/) was used for histograms and heat maps. Cytoscape3.7.1 was used to map the gene–metabolite correlation network. Adobe Illustrator CS6 (https://www.adobe.com/cn/products/illustrator.html) was used for illustration.

## Results

### Sample quality control analysis

By overlapping the base peak ion flow diagrams (BPC diagram) of different QC samples (Supplementary Figure S1), it can be concluded that the metabolite extraction and detection repeatability and instrument stability in this study are good.

Through the detection of blank samples, the residue of substances in the detection process can be investigated (Supplementary Figure S2). In this study, no significant peak was detected in the blank samples, indicating that the substance residue was well controlled and there was no cross-contamination between samples.

### Qualitative and quantitative analysis of *W. cocos* metabolites

The filtered data were identified by the public database and a secondary database built by software (Table 2). The results showed that there were a total of 4909 kinds of positive ion mode (POS), among which there were 4708 known substances, accounting for 95.51% of the positive ions. There were 201 unknown substances, accounting for 4.09% of the positive ions. There were 3310 substances in the negative ion mode (NEG), among which 2883 were known substances (87.10% of the negative ions) and 427 unknown substances (12.90% of the negative ions). The metabolite coverage rate is higher and detection is better when using two ionization methods. In the subsequent analysis process, the data from positive and negative ion modes were analyzed.

**Table 2:** Metabolite identification results.

Type	All	Known	Unknown
POS	4909	4708 (95.51%)	201 (4.09%)
NEG	3310	2883 (87.10%)	427 (12.90%)
Total	8219	7591 (92.36%)	628 (7.64%)

### Multivariate statistical analysis

OPLS-DA and a sorting test were used. OPLS-DA analysis among samples (Supplementary Figure S3) showed significant differences in total metabolites among samples. The sorting test results showed that, in addition to Ld17-VS-Ld34 in NEG, and Hd17-VS-Hd34 and Ld34-VS-Ld51 in POS, the three comparison groups were slightly worse, and the R2' and Q2' of the other comparison groups were lower than the original R2 and Q2, indicating that the analysis results based on OPLS-DA in this study were accurate and reliable.

### Statistical analysis of quantity of differential metabolites

**Figure 1:**
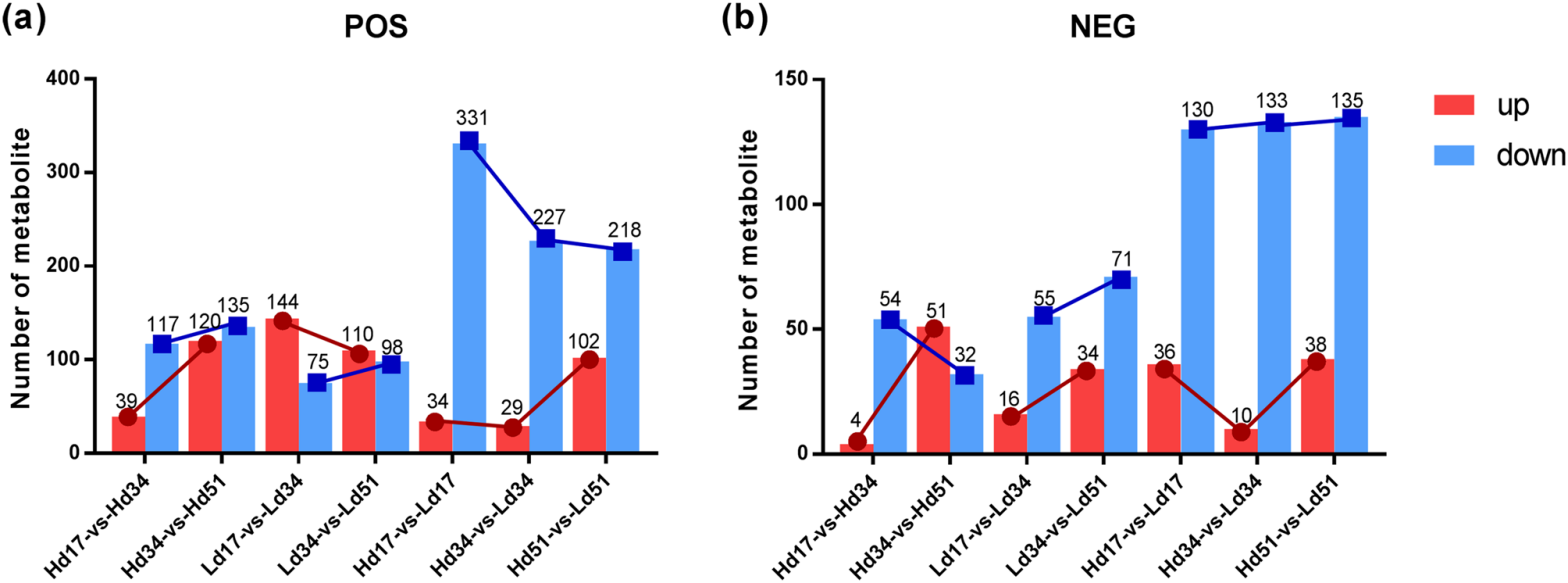
Differential statistics of (**a**) POS metabolites and (**b**) NEG metabolites.

Multivariate statistical analysis of VIP values of OPLS-DA and univariate T-test P values were used to screen the metabolites and assess significant differences between groups. The threshold of significant difference was VIP ≥ 1 and *P* < 0.05. Significant differences in metabolites (Fig. 1; Supplementary Table S1, S2) showed that the metabolites of the two groups at three different culture periods exhibited a higher number of down-regulated metabolites than up-regulated metabolites. In the case of metabolites of POS, the number of down-regulated metabolites reached 331, 227, and 218 at 17, 34, and 51 d, respectively, and the number of down-regulated metabolites decreased with culture time. For metabolites of NEG, the metabolites at 17, 34 and 51 d were 130, 133, and 135, respectively, showing that the metabolites increased with culture time. The number of up-regulated metabolites between the two groups was not high, but they all exhibited similar trends. The up-regulated metabolites (including POS and NEG) at 17, 34, and 51 d showed a trend of decreasing at first and then increasing.

However, the metabolites of the two groups of strains differed from each other in three different culture periods. Among the up-regulated differential metabolites of high-yielding strains (H), there were 39 metabolites of POS and four metabolites of NEG between 17 and 34 d of culture. There were 120 up-regulated differential metabolites of POS between 34 and 51 d of culture, while there were only 51 metabolites of NEG. There was general increasing trend with culture time. Among the down-regulated differential metabolites, POS metabolites increased with culture time, while NEG metabolites decreased as culture time increased.

Among the up-regulated differential metabolites of low-yielding strains (L), 144 were metabolites of POS and only 16 were metabolites of NEG between 17 and 34 d of culture. There were 110 up-regulated differential metabolites of POS between 34 and 51 d of culture, while there were only 34 metabolites of NEG. The number of up-regulated differential metabolites of POS decreased with culture time, while the metabolites of NEG increased with culture time. However, both POS and NEG of the down-regulated differential metabolites increased with increased culture time.

We found that the up-regulated and down-regulated differential metabolites in the H and L group exhibited opposite trends with the extension of culture time, which was consistent with the performance characteristics of the specific metabolites in the two groups.

### KEGG analysis of metabolites

There are 1074 metabolites annotated in the Kyoto Encyclopedia of Genes and Genomes (KEGG) database (Supplementary Table S3), which are distributed in 112 pathways. The maximum number of metabolites noted in metabolic pathway is 594, followed by microbial metabolism in different environments (290), secondary metabolite biosynthesis (259), antibiotic biosynthesis (150), glycerolipid metabolism (90), amino acid biosynthesis (84), and other pathways which all have fewer than 80 metabolites. This sequence is broadly consistent with the transcriptome gene annotation ^22^, except that glycerolipid metabolism in the transcriptome is in 38th position.

**Figure 2:**
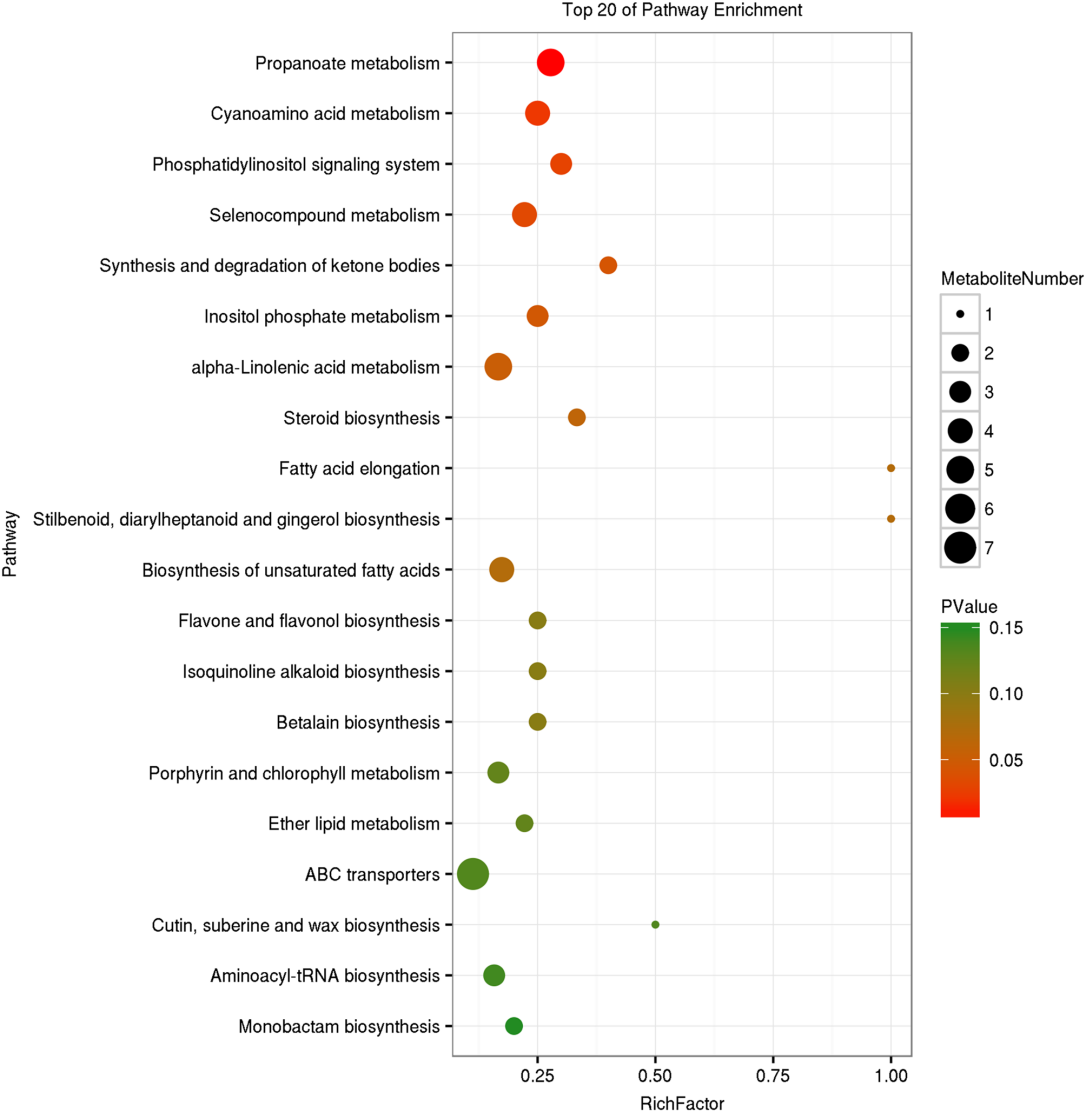
KEGG enrichment map of differential metabolites of two strains after 17 d culture. The vertical axis represents pathway entries, and Rich Factor on the horizontal axis refers to the ratio of the number of differential metabolites in the pathway to the total number of metabolites in the pathway. Higher Rich Factor values indicate a higher degree of enrichment. The size of the circle corresponds to the number of enriched metabolites, with a larger circle indicating more genes. *P* values range from 0 to 1, corresponding to the gradual change from red to green, with red being zero and indicating more significant enrichment. The figure is plotted with the first 20 pathways of *P* values from smallest to largest.

**Figure 3:**
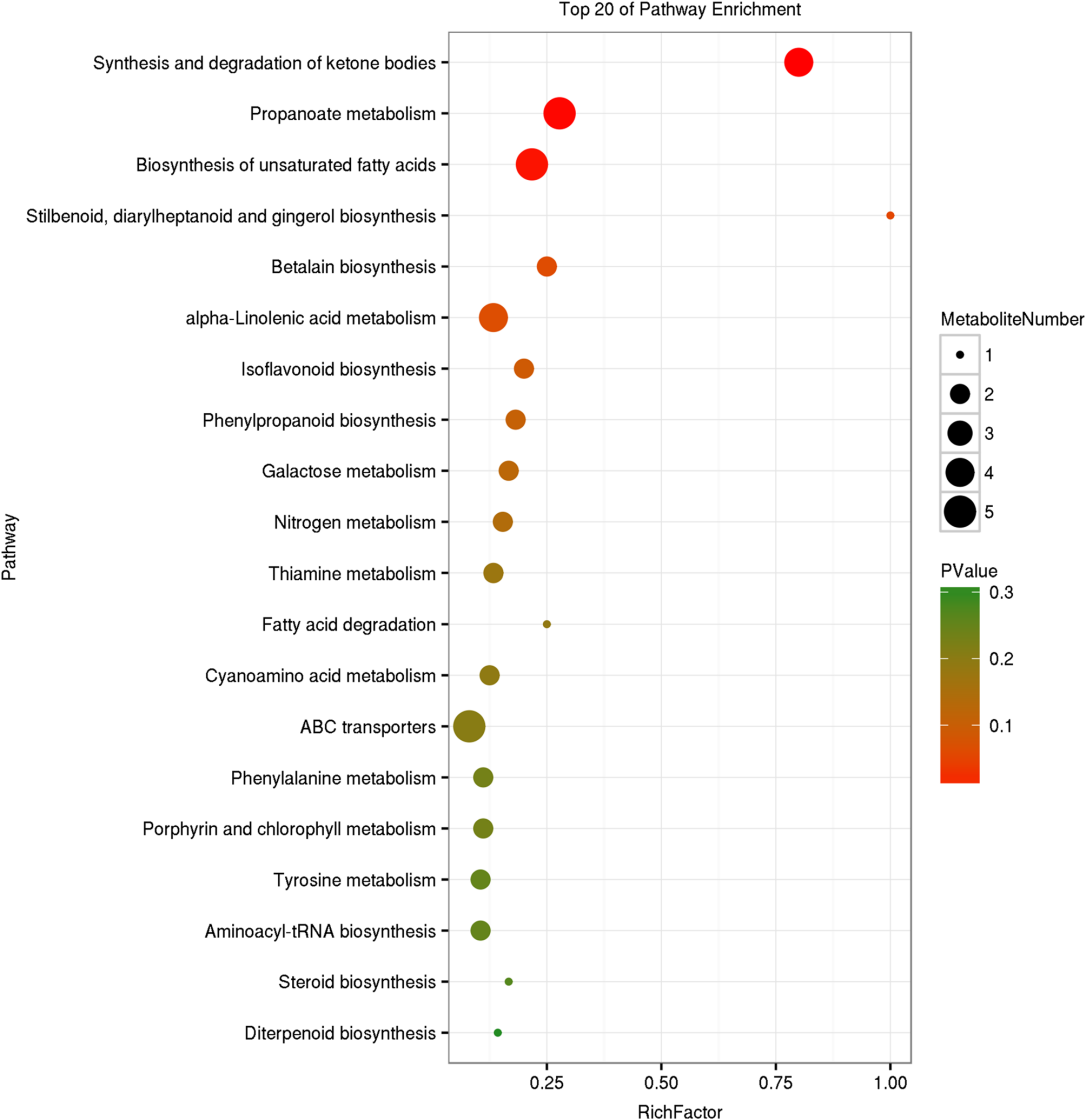
KEGG enrichment map of differential metabolite of two strains at 34 d culture. The vertical axis represents pathway entries, and Rich Factor on the horizontal axis refers to the ratio of the number of differential metabolites in the pathway to the total number of metabolites in the pathway. Higher Rich Factor values indicate a higher degree of enrichment. The size of the circle corresponds to the number of enriched metabolites, with a larger circle indicating more genes. *P* values range from 0 to 1, corresponding to the gradual change from red to green, with red being zero and indicating more significant enrichment. The figure is plotted with the first 20 pathways of P values from smallest to largest.

**Figure 4:**
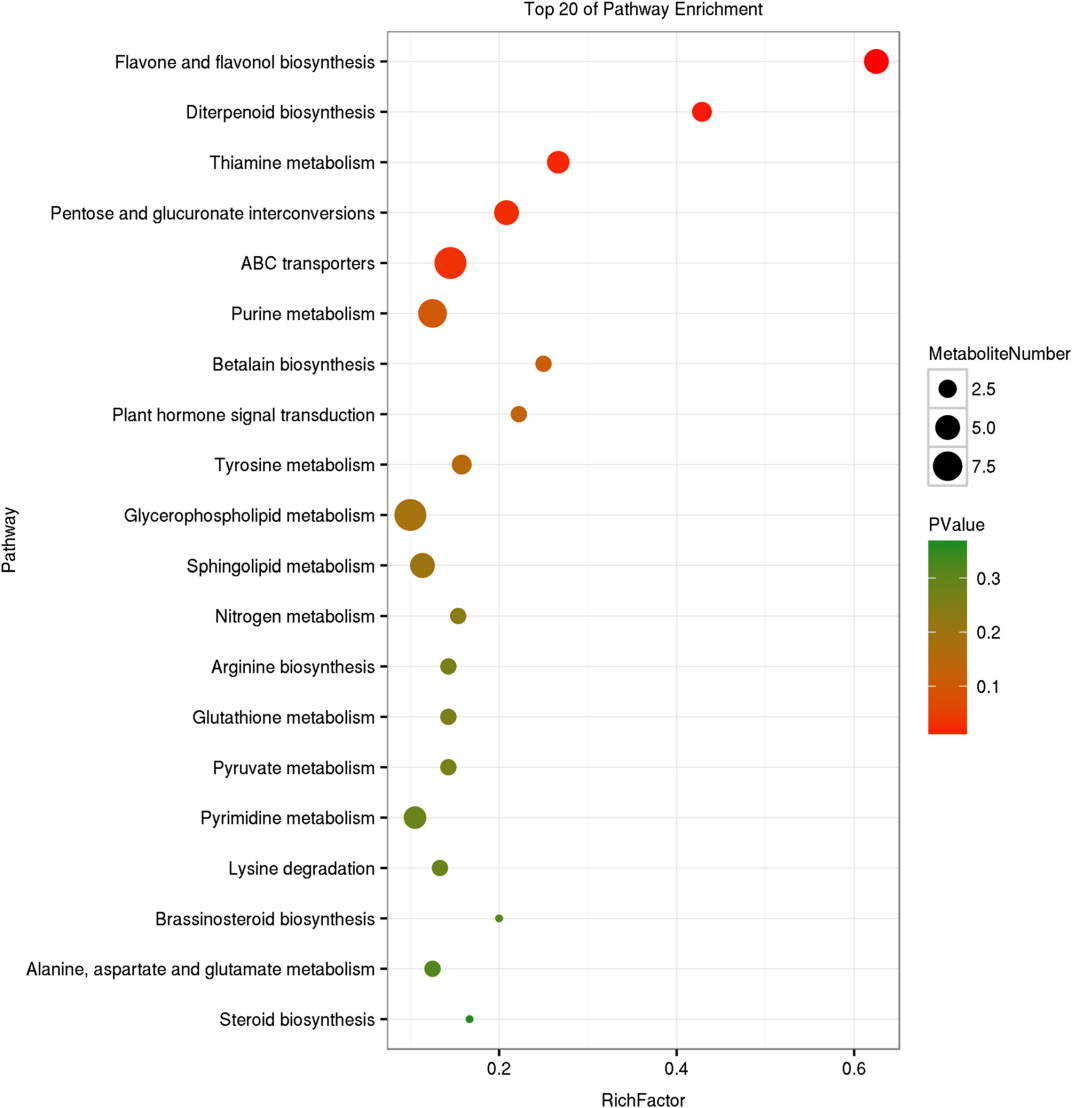
KEGG enrichment map of differential metabolite of two strains at 51 d culture. The vertical axis represents pathway entries, and Rich Factor on the horizontal axis refers to the ratio of the number of differential metabolites in the pathway to the total number of metabolites in the pathway. Higher Rich Factor values indicate a higher degree of enrichment. The size of the circle corresponds to the number of enriched metabolites, with a larger circle indicating more genes. *P* values range from 0 to 1, corresponding to the gradual change from red to green, with red being zero and indicating more significant enrichment. The figure is plotted with the first 20 pathways of *P* values from smallest to largest.

KEGG enrichment analysis of differential metabolites in the two strains (Figs. 2, 3, 4) showed that betalain biosynthesis (other secondary metabolites), steroid biosynthesis (lipid metabolism), and ABC transporters (membrane transport) were enriched pathways in the three different culture periods. The metabolism of α-linolenic acid, acetone, thiamine, tyrosine, cyanoamino acid, nitrogen, porphyrin, and chlorophyll, and biosynthesis of aminoyl-tRNA, unsaturated fatty acids, flavone and flavonol, stilbenoid, and diarylheptanoid and gingerol biosynthesis were enriched in two culture stages, as was the synthesis and degradation of ketone bodies. A variety of amino acid metabolism and lipid metabolism, biosynthesis of diterpenoid, isoflavonoid and isoquinoline alkaloid, and various signal systems were enriched in one culture period. The results of differential metabolites enrichment confirm the results of transcriptome analysis^22^, which indicated that the biosynthesis and accumulation of triterpenoids is closely related to the synthesis of sterols, maintenance of membrane structure, and membrane transport function.

### STEM analysis of metabolites

**Figure 5:**
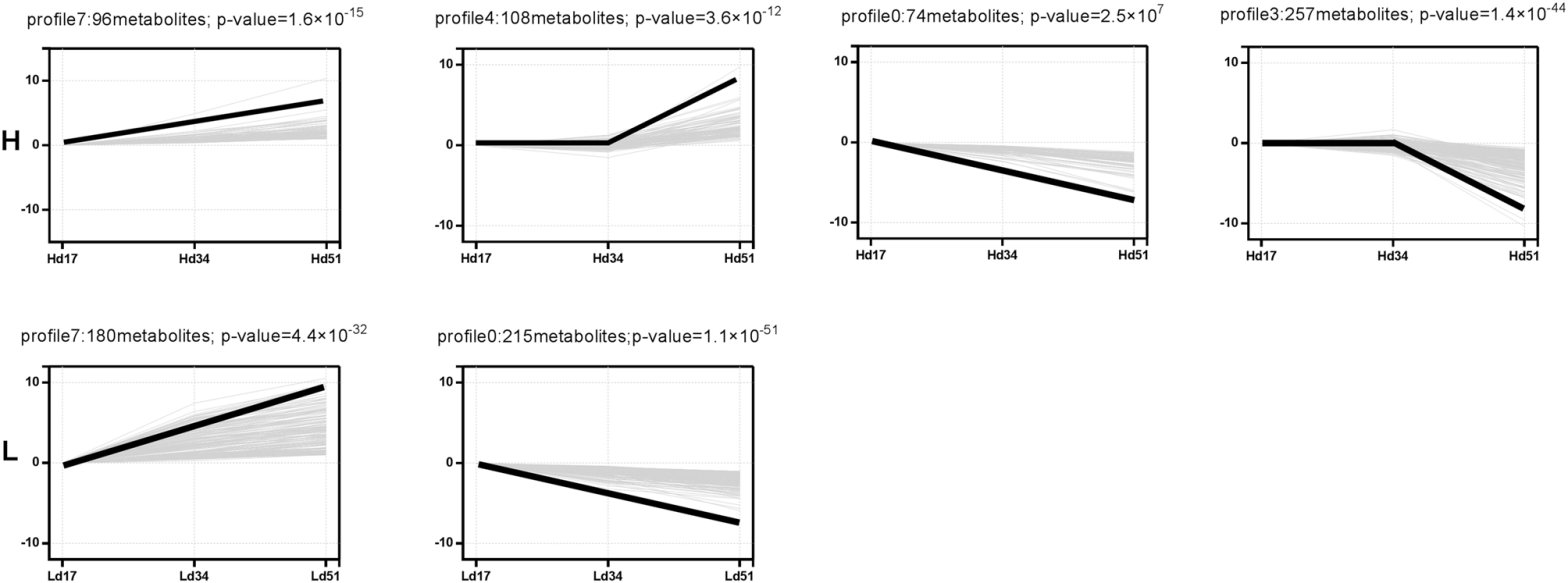
Patterns of metabolite expressions in the two strains. In each box, the thin gray line represents a metabolite expressed in this pattern, and the thick black line represents the expression trend of all metabolites in this pattern. The number of metabolites for this pattern and its P-value are indicated on the pattern box.

Short time-series expression miner (STEM) software was used to analyze metabolite expression patterns for all differential metabolites in the three culture periods in the H and L strains, and eight metabolite expression patterns were obtained from each (Fig. 5). Significant patterns were obtained for four metabolites in the H strain (profiles 0, 3, 4, 7; *P* < 0.001), and the opposite expression patterns were present. Two metabolites exhibited significant patterns in the L strain (profiles 0, 7; *P* < 0.001), which also reflected the existence of different expression patterns of metabolites. The metabolite with significant expression patterns in both strains showed opposing trends of increase and decrease.

To determine the function of metabolites in the significant patterns of the two strains, we performed KEGG enrichment analysis on the metabolites in the six significant patterns (Supplementary Table S4). Isoquinoline biosynthesis was significantly enriched in both the H and L strain, with it showing a decreasing trend in both. Metabolism of different amino acids enriched in the two strains showed a decreasing trend. However, glucose metabolism exhibited a different pattern. In the L strain, the pentose and glucuronate interconversions increased, while fructose and mannose metabolism increased but galactose metabolism decreased in the H strain. The biosynthesis of antibiotics, indole alkaloid biosynthesis, inositol phosphate metabolism, and phosphatidylinositol signal system in the H strain decreased, while diterpenoid biosynthesis, propanoate metabolism, and plant hormone signal transduction exhibited an increasing trend. Although few metabolites were annotated on the KEGG pathway, it can be seen that the biosynthesis and accumulation of triterpenoids are closely related to the metabolism of isoquinoline and sugars.

### Association analysis of differential genes and differential metabolites

**Figure 6:**
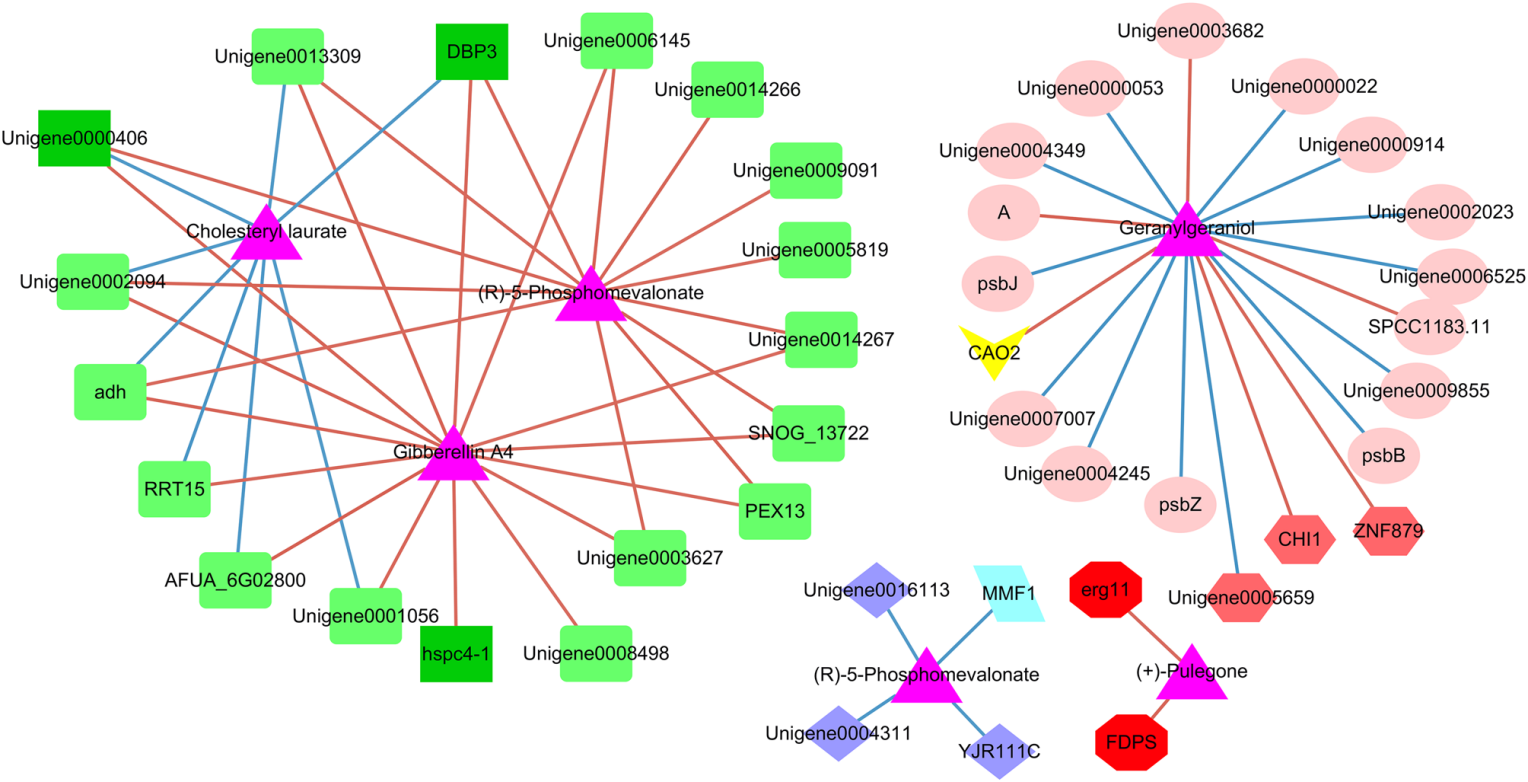
Correlation between DEGs and differential metabolites. Names or number of DEGs and metabolites are marked on the node graph. Magenta triangles represent metabolites; light green boxes represent genes with opposite expressions by STEM analysis; dark green boxes represent regulatory factors and genes with opposite expressions; dark red hexagons represent regulatory factor genes; light blue parallelograms represent regulatory genes; bright red octagons represent regulatory genes in related pathways; yellow V shapes represent genes in related pathways of triterpenoid synthesis. Red lines indicate a positive correlation, while blue lines indicate a negative correlation.

To filter out genes closely related to triterpenoid biosynthesis in *W. cocos*, we calculated correlation coefficients between different metabolites and differentially expressed genes (DEGs)^22^. The threshold value for gene and metabolite pair screening was | r |< 0.9, *P* < 0.01 (Fig. 6; Supplementary Table S5). The results were divided into the following four categories: Genes closely related to cholesteryl laurate (NEG00045), (R)-5-phosphomevalonate (POS04730), and gibberellin A4 (NEG00066); genes closely related to geranylgeraniol (POS00077); genes closely related to (R)-5-phosphomevalonate (POS04731); and genes closely related to ( +)-pulegone (POS00001).

Lanosterol 14-alpha-demethylase (*erg11*) (Unigene0015621) and farnesyl-diphosphate synthase (*FDPS*) (Unigene0002741), which are regulatory genes (Plant Transcription Factor Database) (http://plntfdb.bio.uni-potsdam.de/v3.0/), were both closely positively correlated with (+)-pulegone in the triterpenoid biosynthesis pathway. *FDPS* (Unigene0002741) is a terpenoid backbone biosynthesis pathway gene. *Erg11* (Unigene0015621), which is a steroid biosynthesis pathway gene, was closely correlated with the core genes of the opposite expression trend in the two strains^22^. Carotenoid oxygenase (*CAO2*) (Unigene0011353), a carotenoid biosynthesis pathway gene, was closely positively correlated with geranylgeraniol in the diterpenoid biosynthesis pathway.

Among the genes closely related to geranylgeraniol, six (*CAO2*, *CHI1*, *ZnF879*, *SPCC1183.11*, etc.) belong to positive regulator genes, and 13 (*PSBJ*, *PSBZ*, *PSBB*, etc.) belong to negative regulator genes. *MMF1* is a regulatory gene among the genes that is closely negatively correlated with (R)-5-phosphomevalonate in the terpenoid backbone biosynthesis pathway.

The genes closely related to cholesteryl laurate, (R)-5-phosphomevalonate, and gibberellin A4 all exhibit opposing expression trends in time series between the two groups. *DBp3*, GroES-like protein (*adh*), unigene0002094, unigene0013309, and unigene0000406 were closely positively correlated with (R)-5-phosphomevalonate and gibberellin A4, but were negatively correlated with cholesteryl laurate synthesis. DBp3 and unigene0000406 are regulatory genes. RRT15, AFUA_6G02800, and unigene0001056 were closely positively correlated with gibberellin A4 and negatively correlated with cholesteryl laurate synthesis. Unigene0006145, unigene0014267, unigene0003627, *SNOG_13722*, and *PEX13* were closely positively correlated with (R)-5-phosphomevalonate and gibberellin A4. Unigene0009091, unigene0014266, and unigene0005819 were closely positively correlated with (R)-5-phosphomevalonate, while unigene0008498 and hspc4-1 were closely positively correlated with gibberellin A4. The network relationship between multiple genes and cholesteryl laurate, (R)-5-phosphomevalonate, and gibberellin A4 reflects the complexity of secondary metabolism regulation.

## Discussion

In this study, two ionization methods were adopted and a total of 8219 positive and negative ions were detected, among which 7591 substances were known, accounting for 92.36% of the total. The sample quality control analysis included the BPC superposition chart of QC sample essential spectrum detection and the blank sample essential spectrum detection chart, which together indicated that the metabolite extraction and detection in this study had good repeatability and instrument stability, the substance residue was well controlled, and there was no cross-contamination between samples. The results of OPLS-DA analysis were therefore accurate and reliable after a sorting test.

By combining the VIP value of OPLS-DA and the T-test P value, metabolites that exhibited significant differences between groups could be screened. The results showed that the number of down-regulated significant differential metabolites was higher than the number of up-regulated metabolites in the two strains at three different culture periods. The number of up-regulated metabolites (including POS and NEG) in the two strains was not high, but all showed a pattern of decreasing first and then increasing. In the H strain, the up-regulated metabolites increased along with culture time. In down-regulated metabolites, the POS metabolites increased along with culture time, while NEG metabolites decreased. In the L strain, down-regulated differential metabolites increased with culture time. The up-regulated metabolites and POS metabolites decreased along with culture time, while the NEG metabolites increased. These results are consistent with the performance characteristics of the differences found in specific metabolites across the two groups.

Substances of triterpenoids do not meet the criteria of different express metabolites. The contents of total triterpenoids were highly significantly different between high-yielding and low-yielding strains at three different culture periods^27^. In significant differential metabolites, the number of down-regulated metabolites was higher than up-regulated metabolites. These results indicate that triterpenoids, as secondary metabolites, have a small content and a wide variety of species, and the yield of each substance is small and affected by various factors. But their cumulative effect cannot be ignored.

The results of KEGG annotation of metabolites were broadly consistent with those of transcriptome gene annotation. KEGG enrichment results indicated that metabolites were mainly enriched in steroid biosynthesis (lipid metabolism) and ABC transport (membrane transport), which confirmed the results of transcriptome analysis. The biosynthesis and accumulation of triterpenoids are closely related to sterol synthesis, maintenance of the membrane structure, and membrane transport function.

STEM analysis of metabolites showed that there was a significant difference in the accumulation of metabolites between the two strains. The pentose and glucuronate interconversions increased in the L strain. In the H strain, fructose and mannose metabolism increased but galactose metabolism decreased. These results indicate that there were significant differences not only in the synthesis and accumulation of triterpenoids in phenotypes, but also in glucose metabolism between the two strains. The pentose and glucuronate interconversions in the L strain showed an increasing trend, suggesting that the synthesis and accumulation of polysaccharides might be higher than in the H strain. It was also found in the culture process of the two strains that the mycelial growth in the L strain was significantly higher than in the H strain. Two main active components of *W. cocos* are triterpenoids and polysaccharides, so in this study we speculated that as the synthesis and accumulation of either triterpenoids or polysaccharides changed in *W. cocos*, the other would exhibit an opposing change trend.

The association analysis of DEGs and differential metabolites between the two strains showed that there were three DEGs closely related to the differential metabolites, namely, *erg11* (Unigene0015621), *FDPS* (Unigene0002741), and *CAO2* (Unigene0011353), which are annotated into pathways related to triterpenoid synthesis.

*FDPS* catalyzes the condensation dimethylallyl diphosphate (DMAP) with two units of isopentenyl pyrophosphate (IPP) to produce farnesyl pyrophosphate (FPP), a key intermediate located at the branch point in the mevalonate (MVA) pathway^28^. This reaction is considered to be a rate-limiting step in isoprene biosynthesis. In addition to being used directly for protein farnesylation, the FPP molecule is used as backbone for the synthesis of other sesquiterpenoids and as a building block in the biosynthesis of larger terpenoids such as diterpenes, dolichols, mitochondrial ubiquinones, and sterols. In most organisms, the main end products derived from FPP are sterols including ergosterol and cholesterol. Farnesyl-diphosphate synthase (*FPS*) is encoded by a variable number of copies of associated genes. Plants typically have two copies of *FPS*, with alternate splicing producing up to three different isoforms whose expression varies as a function of location, development, and environmental conditionss^29^. *Arabidopsis thaliana* contains at least two genes encoding *FDPS* (*FPS1* and *FPS2*), which have different patterns of expression^30^. The content of triterpenoids in *Sanghuangporus baumii* was correlated with the transcription level of *FPS* at different developmental stages. In combination with our results of previous studies ^22^, there were three genes annotated as *FDPs*, and their expression patterns differed. One was highly expressed in the L strain, while the expression of the other two genes did not reach a significant difference. We suggest that the low triterpenoid accumulation in the L strain may not be due to the low expression of the gene in the direction of triterpenoid synthesis, but may be due to the fact that the catalytic products of the high-expression gene do not flow to the direction of triterpenoid synthesis. The results of this study are not consistent with those of previous studies. This may be because the *FDPS* genes annotated at the same time catalyzed different products. It may be that the overexpressed *FDPS* genes of previous studies catalyzed products in the direction of triterpenoid synthesis.

*Erg11* (also known by its gene name CYP51A1) is found in all biological kingdoms and is considered the most ancient cytochrome P450^31^. According to the Plant Transcription Factor Database, *erg11* (Unigene0015621) is a regulatory gene. *Erg11* is a drug target in infectious diseases such as fungal infections^32^. It has important biological consequences within the sterol synthesis pathway^33^. STEM analysis of the transcriptome showed that it was closely negatively correlated with the core gene Pm20d2 outside the pathway^22^. In this study, *erg11* (Unigene0015621) was highly expressed in the L strain, suggesting that lanosterol was largely diverted to sterol synthesis in low-yielding strains. Three other genes annotated as *erg11* did not show significant differences. In this study, *erg11* (Unigene0015621) was significantly correlated with ( +)-pulegone, which is a monoterpenoid product synthesized from the shunt of geranyl diphosphate catalyzed by *FDPS*. Therefore, *erg11* not only plays an important role in shunting the products of lanosterol but also affects the shunting of the upstream geranyl diphosphate, which is a key enzyme that has important functions.

*CAO2* is a gene in the carotenoid biosynthetic pathway which catalyzes torulene (a C(40) carotene) to β-Apo-4 '-carotenal^34^. In this study, it was closely positively correlated with the diterpene geranylgeraniol, which indicated that there are not only monoterpenoids, but also diterpenoids, in the shunt of geranyl diphosphate. Metabolome analysis showed that the *FDPS* gene catalytic products may flow to other synthetic pathways in the L strain, reducing the flow of precursor substances to triterpenoids, which explains the low accumulation of triterpenoids in the L strain.

In summary, the core genes related to triterpenoid synthesis and accumulation confirmed by metabolome analysis are *erg11* (Unigene0015621) and *FDPS* (Unigene0002741). Metabolome analysis confirmed that the biosynthesis and accumulation of triterpenoids are closely related to the synthesis of sterols, membrane structure maintenance, and membrane transport function. Perhaps the low-yielding strain of triterpenoids was the high-yielding strain of polysaccharides. We speculate that the synthesis and accumulation of triterpenoids and polysaccharides in *W. cocos* exhibits opposing trends (as one increases the other decreases). The high level of triterpenoids in the H strain may be not only the result of the high expression of some genes in the H, but also the result of the high expression of some genes in the L strain flowing to other pathways and diverting the precursor substances of triterpenoids, which resulted in a decrease in the synthesis and accumulation of triterpenoids. In order to improve the synthesis and accumulation of a certain class of substances, we should not only pay attention to the genes with high expression in the H strain, but also to the genes with high expression of shunt precursor substances in the L strain, as controlling their expression may increase the synthesis of the required substances.

## Supplementary Information

Supplementary Tables.

Supplementary Legends.

Supplementary Figure 1.

Supplementary Figure 2.

Supplementary Figure 3.
