# Supplementary material for: Metabolome analysis of key genes for synthesis and accumulation of triterpenoids in Wolfiporia cocos
Source: Sci Rep. 2022 Jan 28;12:1574. doi: 10.1038/s41598-022-05610-3 (PMC8799705; doi:10.1038/s41598-022-05610-3)
Supplement: Supplementary file 2 — Supplementary Legends. [file 41598_2022_5610_MOESM2_ESM.pdf]

Figure S1 BPC overlay chart of QC sample mass spectrometric detection. (a) NEG mass spectrometric detection; (b) POS mass spectrometric detection.

Figure S2 Mass spectrogram detection of blank sample. (a) NEG mass spectrogram detection of blank sample; (b) POS mass spectrogram detection of blank sample.

Figure S3 OPLS-DA and sorting inspection. In turn, a-i is Hd17-vs-Ld17，Hd34-vs-Ld34，Hd51-vs-Ld51，Hd17-vs-Hd34，Hd34-vs-Hd51，Hd17-vs-Hd51，Ld17-vs-Ld34，Ld34-vs-Ld51，Ld17-vs-Ld51; capital letters represent OPLS-DA, lowercase letters represent sorting inspection; red letters represent NEG, blue letters represent POS.
