# Supplementary figures and images for: Metabolome analysis of key genes for synthesis and accumulation of triterpenoids in Wolfiporia cocos
Source: Sci Rep. 2022 Jan 28;12:1574. doi: 10.1038/s41598-022-05610-3 (PMC8799705; doi:10.1038/s41598-022-05610-3)

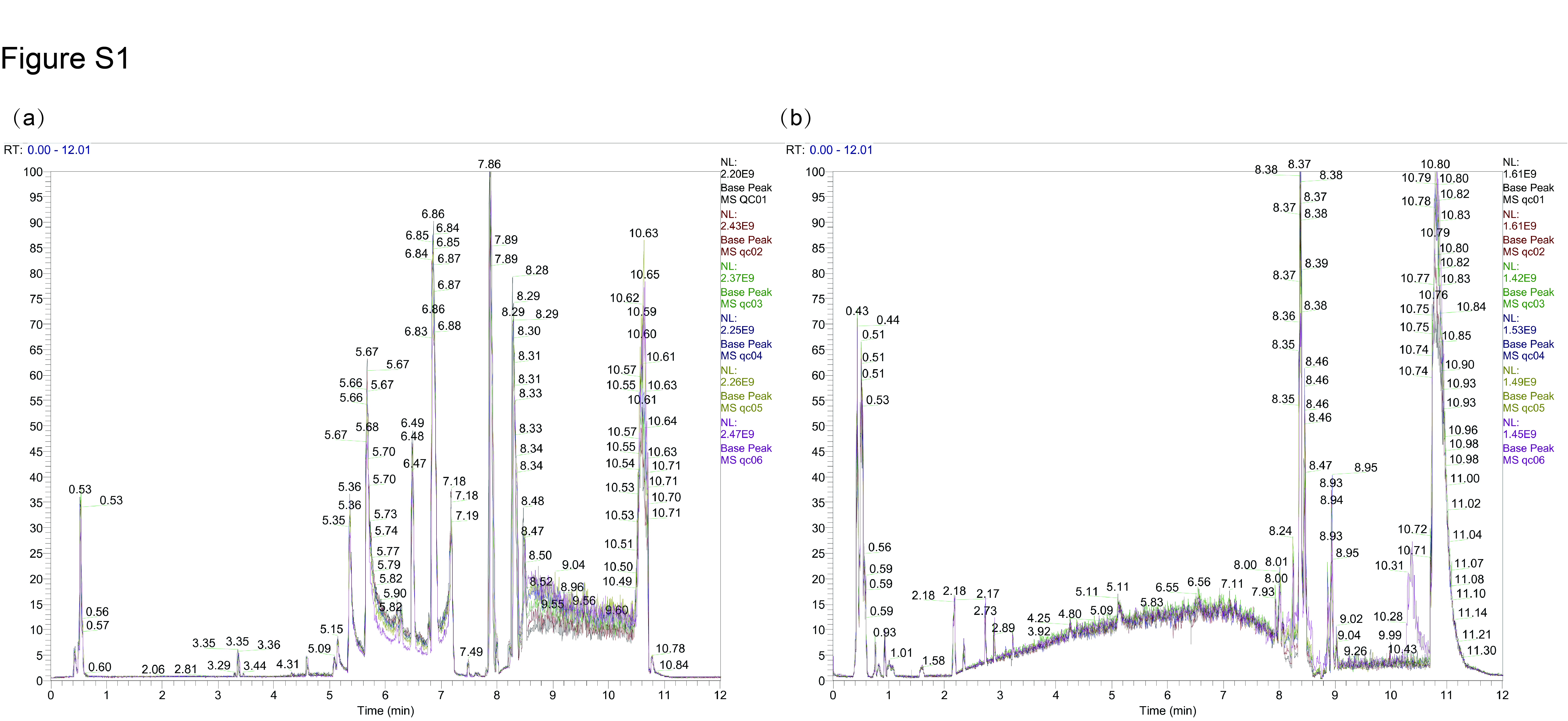

Supplement: Supplementary file 3 — Supplementary Figure 1. [file 41598_2022_5610_MOESM3_ESM.tif]

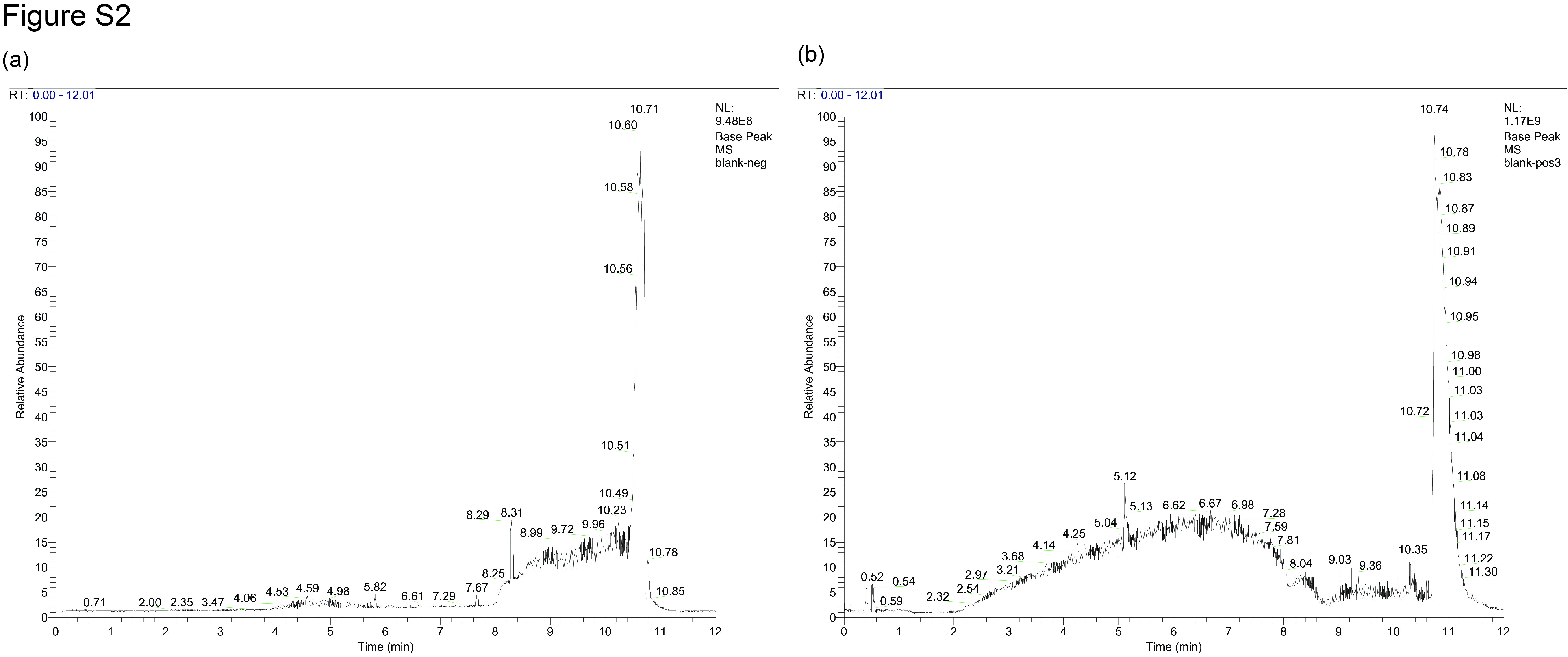

Supplement: Supplementary file 4 — Supplementary Figure 2. [file 41598_2022_5610_MOESM4_ESM.tif]
